# Inflammatory Myofibroblastic Tumor Presenting with Diabetes Insipidus in an Eight-Year-Old Boy: A Case Report

**DOI:** 10.4274/jcrpe.1961

**Published:** 2015-12-03

**Authors:** Erkan Sarı, Erman Ataş, Engin Burak Bulut, Sebahattin Sarı, Onur Akın, Mehmet Saldır, Yıldırım Karslıoğlu, Ediz Yeşilkaya

**Affiliations:** 1 Gülhane Military Medicine Academy, Department of Pediatric Endocrinology, Ankara, Turkey; 2 Gülhane Military Medicine Academy, Department of Pediatric Oncology, Ankara, Turkey; 3 Gülhane Military Medicine Academy, Department of Pediatric Surgery, Ankara, Turkey; 4 Gülhane Military Medicine Academy, Department of Radiology, Ankara, Turkey; 5 Gülhane Military Medicine Academy, Department of Pathology, Ankara, Turkey

**Keywords:** Inflammatory myofibroblastic tumor, diabetes insipidus, child

## Abstract

Inflammatory myofibroblastic tumors (IMT) develop as a non-neoplastic proliferation of myofibroblasts in a myxoid to collagenous stroma admixed with inflammatory cells. The symptoms depend on the specific location of the tumor, which can be anywhere, but is particularly in the respiratory system. Thus, patients with IMT can present with a variety of findings. A pediatric patient with IMT who presented with cough, breathlessness, polyuria-polydipsia, and convulsions is described in this report.

**WHAT IS ALREADY KNOWN ON THIS TOPIC?**

Tumors may cause central diabetes insipidus.

**WHAT THIS STUDY ADDS?**

Inflammatory myofibroblastic tumor is a rarely seen tumor and may cause central diabetes insipidus. Any finding associated with idiopathic central diabetes insipidus should be investigated thoroughly.

## INTRODUCTION

Inflammatory myofibroblastic tumor (IMT), (also known as plasma cell granuloma, inflammatory myofibrohistiocytic proliferation, inflammatory pseudotumor, cellular inflammatory pseudotumor) is a non-neoplastic proliferation of myofibroblasts in a myxoid to collagenous stroma admixed with inflammatory cells (1,2). Although pulmonary system is the most frequently involved site, central nervous system (CNS), gastrointestinal tract, tonsils, urogenital tract, heart, and orbit may also be affected (3,4). Concomitant involvement, such as that of the lung and central nervous system, might be encountered as well. These tumors, with their mass effect, have a primary role in the etiology of central diabetes insipidus (cDI). Here, we report an 8-year-old boy who had IMT and who presented with convulsions and polyuria-polydipsia in addition to his respiratory symptoms.

## CASE REPORT

**Figure 1:**
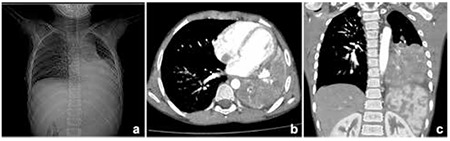
Postero-anterior chest radiography showing a huge area of radioopacity on the left middle and lower hemithorax and calcification in the central area of the opacity (a). Contrast-enhanced arterial phase axial (b) and multiplanar-reformatted coronal (c) chest computed tomography images scan clearly show a giant, calcified soft tissue mass in the left lower hemithorax. Contrast-enhanced axial computed tomography shows heterogeneous enhancement and obscured border between the mass and the heart in some areas, a finding consistent with pericardial involvement

This 8-year-old male patient had been suffering from recurrent cough, breathlessness, and malaise since he was 5 years old. He had been administered antibiotics orally for tonsillitis almost every 1-2 months for his complaints. These episodes had been usually associated with fever. He also had a history of five episodes of afebrile convulsions with temporary vision loss. Although laboratory results were negative for tuberculosis, he was administered antituberculosis therapy for one year because of the opacity noted in his chest roentgenogram (Figure 1a). However, he did not respond to the therapy. Also, he had been suffering from polyuria and polydipsia for almost three years. He has been drinking water (6 L/m2/day) and waking up approximately three times to drink and urinate each night.

At the time of admission, the patient’s vital functions were within normal ranges. Physical examination revealed a thin child who weighed 23 kg (15th percentile) and measured 115 cm in height (30th percentile) but appeared to be healthy. His oral mucosa was dry and breath sounds were reduced on the left side. Other physical findings were normal including the neurological system.

**Figure 2:**
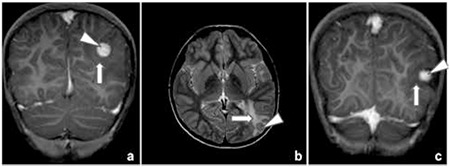
Coronal contrast-enhanced T1-weighted (a) and axial T2-weighted (b, c) magnetic resonance image shows two homogeneously enhancing lesions with surrounding vasogenic edema in the cortical-subcortical region of the left parietal and temporo-occipital lobes

**Figure 3:**
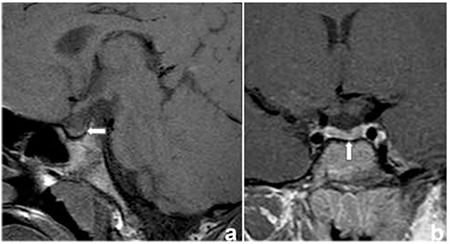
Sagittal pre-contrast (a) and coronal post-contrast (b) T1-weighted magnetic resonance images showing a small adenohypophysis and decreased normal hyperintense signal of the neurohypophysis

Laboratory results showed a hemoglobin level of 11.7 g/dL, a total leucocyte count of 10.800/mm3, and a differential count within normal ranges. While the erythrocyte sedimentation rate (82 mm/h) and C-reactive protein (38 mg/L) were elevated, the electrolytes, serum calcium, uric acid, as well as renal and liver functions were within normal ranges. Basal serum sodium, urea, and glucose levels were also within normal ranges (142 meq/L, 11 mg/dL, and 96 mg/dL, respectively). Before the water deprivation test, urine specific gravity, serum and urine osmolarity values were 1,000, 293 mOsm/kg, and 133 mOsm/kg, respectively. Following a 14-hour period of water deprivation, the serum sodium level increased to 155 meq/L and urine specific gravity, serum and urine osmolality were found as 1,007, 323 mOsm/kg and 240 mOsm/kg, respectively. Serum antidiuretic hormone was <0.5 pg/mL both before and after the water deprivation test and these levels were consistent with cDI. Polyuria and polydipsia symptoms were relieved with desmopressin. The pituitary hormone profile was in normal ranges except for a low level of insulin-like growth factor of 79 ng/dL (-2/-3 SDS) and antidiuretic hormone. CNS investigation revealed presence of two 10- and 14-mm lesions on the left parietal and temporo-occipital lobes and a small adenohypophysis (2 mm in diameter). The infundibulum was normal in appearance, but neurohypophysis intensity was decreased (Figure 2, 3).

**Figure 4:**
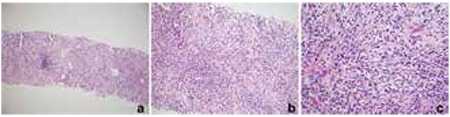
The tumor, infiltrating and replacing the lung parenchyma is seen in low magnification (a). Higher magnification powers revealed a proliferation of cells with bland-looking oval to fusiform-shaped nuclei on a background with heavy inflammatory cell infiltration composed of neutrophils, eosinophils, lymphocytes, plasma cells, and histiocytes (b, c)

Contrast-enhanced computed tomography of the chest showed a large lobulated calcified mass (Figure 1b, 1c). The lung biopsy gave no morphological clues of malignancy, such as increased and/or abnormal mitotic activity, conspicuous cellular atypia, or necrosis (Figure 4). Immunohistochemically, the proliferating cells were highlighted with vimentin, smooth muscle actin, and muscle-specific actin. Focal and weak immunoreactivity against desmin was also noted in some areas. There was no nuclear staining with anaplastic lymphoma kinase (ALK). These morphological findings were interpreted to be in favor of IMT. Later, a thorough examination of the resection material also confirmed the diagnosis of IMT.

Clinical and pathological findings suggested IMT. The mass was excised with an incision extending from the diaphragm to the pericardium, and the invaded part in the atrium was trimmed. After an uneventful pneumonectomy, the patient was discharged. Corticosteroid or chemotherapy was not considered because of the unclear benefits. Three months after pneumonectomy, the patient’s acute phase reactants decreased to normal levels and the febrile episodes diminished, due to resection of the inflammatory focus. A contrast-enhanced magnetic resonance scan performed three months after the pneumonectomy showed findings similar to the former magnetic resonance image, with two well-defined round lesions with perifocal edema.

Currently, the patient is being followed with regard to his growth velocity, potential adenohypophysis hormone deficiency, as well as with regard to cerebral lesions.

## DISCUSSION

The World Health Organization defined IMT in 1994 as tumors characterized by absence of anaplasia, intermixture of lymphocytes and plasma cells among spindle cells, and paucity of mitotic cells (5). Although the etiology of IMT is unknown, these tumors may have some associations with certain microorganisms such as the Epstein-Barr virus or the human herpes virus. IMT mainly affects children and young adults (6). It can occur anywhere in the body (7). Although most of the IMT are isolated and associated with the pulmonary system, very rare, distant secondary involvement has been reported due to the multifocal nature of the lesion rather than metastatic spread (8). The clinical presentation depends on the site and extent of involvement, but systemic symptoms such as fever, malaise, and weight loss can be seen in 15-30% of patients (9).

Intracranial IMT is seen rarely, and can be isolated or multifocal. Major complaints are headache, seizure, and visual disturbances (2). Isolated sellar IMT has also been reported in these patients but was confirmed histopathologically in only one patient. This case was diagnosed as IMT after four years of follow-up with a diagnosis of cDI. Pituitary gland magnetic resonance imaging was normal at the time of diagnosis of cDI (10). A combination of pulmonary and intracranial IMTs have been reported in a small number of cases (2,11,12,13).

Acquired cDI is a rare entity, and radiological investigations have shown that most of these cases are associated with entities such as craniopharyngioma, germinoma, and histiocytosis. However, this association cannot always be shown in the early stages and, therefore, it is suggested that patients with idiopathic cDI need to be followed up. DI associated with IMT is a rare and not well understood concomitance (2,14,15). DI develops as a result of a pituitary pathology, but it may also be associated with hypothalamic involvement. In our patient also, because we have no radiologic or histopathologic data about the exact site, the DI may be due to IMT in the pituitary gland or in the hypothalamus. The findings of our patient demonstrate that cases with cDI should be investigated systematically.

Complete surgical resection, if possible, is the best treatment modality for IMT, but aggressive resection should be avoided due to the local invasive nature of these lesions. The indeterminate biological behavior of these tumors require continuous monitoring. For unresectable tumors, radiotherapy, chemotherapy, or anti-inflammatory drugs are alternative treatments. Crizotinib has also been reported to yield promising results in patients with ALK-positive lesions (7).

It is difficult to diagnose IMT because it is a rare entity and also because it may be associated with different organ system symptoms as seen in granulomatous diseases like Wegener, sarcoidosis, etc. A possible diagnosis of IMT needs to be considered in children with DI and respiratory problems.
